# The Crystal Structure of the SV40 T-Antigen Origin Binding Domain in Complex with DNA

**DOI:** 10.1371/journal.pbio.0050023

**Published:** 2007-01-23

**Authors:** Gretchen Meinke, Paul Phelan, Stephanie Moine, Elena Bochkareva, Alexey Bochkarev, Peter A Bullock, Andrew Bohm

**Affiliations:** 1 Department of Biochemistry, School of Medicine, and the Sackler School of Graduate Biomedical Sciences, Tufts University, Boston, Massachusetts, United States of America; 2 Banting and Best Department of Medical Research, University of Toronto, Toronto, Ontario, Canada; University of Wisconsin Madison, United States of America

## Abstract

DNA replication is initiated upon binding of “initiators” to origins of replication. In simian virus 40 (SV40), the core origin contains four pentanucleotide binding sites organized as pairs of inverted repeats. Here we describe the crystal structures of the origin binding domain (obd) of the SV40 large T-antigen (T-ag) both with and without a subfragment of origin-containing DNA. In the co-structure, two T-ag obds are oriented in a head-to-head fashion on the same face of the DNA, and each T-ag obd engages the major groove. Although the obds are very close to each other when bound to this DNA target, they do not contact one another. These data provide a high-resolution structural model that explains site-specific binding to the origin and suggests how these interactions help direct the oligomerization events that culminate in assembly of the helicase-active dodecameric complex of T-ag.

## Author Summary

How DNA replicates is a critical question for understanding life. DNA replication remains difficult to investigate in eukaryotes, where it involves a complex, multi-protein apparatus which initiates replication at multiple poorly-defined DNA sequences. This process is far easier to study in viral systems, where the DNA sequences at the origin of replication are well-defined and only one or two proteins are required to initiate replication. In simian virus 40 (SV40), the large T-antigen protein (T-ag) is responsible for recognizing DNA sequences required to start replication, called the origin of replication. SV40 T-ag can also cause DNA to melt or unwind. We report here the crystal structure of the DNA-binding domain of SV40 T-ag on a DNA fragment derived from the viral origin of replication. The structure shows that although T-ag and its functionally analogous protein, papilloma virus E1, share no detectable sequence homology in this region, the two domains bind the DNA in similar ways. In both cases, DNA binding is thought to initiate assembly of a complex of the full-length proteins on DNA. Interestingly, SV40 T-ag DNA-binding domains do not interact with one another when bound to DNA. In addition to describing the molecular details of the DNA–protein interactions and the alterations in protein structure induced by DNA binding, we present a model describing the subsequent assembly events.

## Abstract 3

Crystal structures of the SV40 large T antigen (T-ag) origin binding domain (obd) with and without DNA show how two T-ag obds orient head-to-head and engage with the major groove.

## Introduction

Viral DNA replication involves a sequence of carefully orchestrated steps including recognition of the origin by a protein (the initiator) or proteins, melting of the origin DNA, replication protein A (RPA)-dependent unwinding of the DNA, and recruitment of polymerase and other replication factors (for reviews, see [1–3]). Study of this process in eukaryotes has been hampered by uncertainty regarding the eukaryotic origin sequences and by the complexity of the proteins involved in eukaryotic origin recognition. While origin sequences have been identified for Saccharomyces cerevisiae, they are not yet identified in the genomes of higher eukaryotes [4,5]. In contrast, replication of small DNA tumor viruses such as SV40 and papilloma virus involves well-defined origin sequences and requires far fewer proteins for formation of the preinitiation complex. In the case of SV40, a single virally encoded initiator, large T-antigen (T-ag), can bind the SV40 origin, assemble as a set of two hexameric rings, and cause local distortions (ie, melting) of the DNA [6]. In the presence of the single-stranded binding protein (SSB) human RPA [6], SV40 T-ag also unwinds origin containing DNA. Once assembled on the origin, SV40 T-ag also recruits host machinery to replicate the viral DNA (for reviews, see [1–3]).

**Figure 1:**
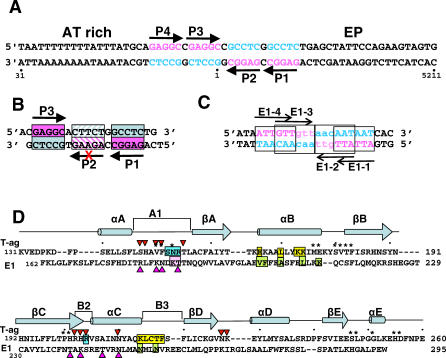
DNA and T-ag Sequences (A) The SV40 64-bp core-origin sequence. The pentanucleotides P1 through P4 are indicated above the sequence. Each GAGGC sequence is colored magenta, and its complement is cyan. The arrows indicate the 5′ → 3′ direction of the pentanucleotide sequence GAGGC. The AT-rich and early palindrome regions of the SV40 core origin are labeled. (B) The DNA duplex used in crystallization of the T-ag obd–DNA complex. This 21-mer contains the palindromic binding sites P1 and P3 and a mutated pentamer P2 site. The GAGGC sequences and their complements are indicated by magenta and cyan boxes, respectively. The altered P2 pentamer is indicated by hash-marks in magenta and cyan. As above, the arrows indicate the 5′ → 3′ direction of the pentanucleotide sequences, and the red X indicates that the P2 sequence is altered. (C) The BPV origin shows the E1 binding sites termed E1–1 through E1–4. The E1 binding sites are imperfect 5′-ATTGTT-3′ hexameric sequences. Boxes outline each binding site, and the binding sites are labeled. The arrows indicate the 5′ → 3′ direction of the binding site. The direct repeats (sites E1–1 and E1–2 or sites E1–3 and E1–4) overlap by 3 bp. The ATTGTT sequence (magenta) and its complement (cyan) are indicated. Lowercase letters are used for the portion of binding sites that do not overlap. (D) Structure-based sequence alignment of SV40 T-ag obd with BPV E1 obd. The secondary structure elements of the T-ag obd are shown above its amino acid sequence. Every tenth residue is indicated with a dot. T-ag obd residues that make base-specific contacts in the DNA co-structure are indicated by cyan boxes. T-ag obd residues that make phosphate interactions are indicated by red triangles above the amino acid sequence. There are two types of T-ag obd–obd interactions: the “head-to-head” type seen in the disulfide-linked dimer (possibly important in double-hexamer formation), and the “side-to-side” type (important in single-hexamer formation) seen in the spiral hexamer. T-ag obd residues that form the protein–protein interface in the disulfide-linked dimer structure are indicated by yellow boxes. T-ag obd residues that comprise the protein–protein interface in the spiral hexamer are indicated by an asterisk (*). Residues for BPV E1 obd that make base-specific contacts or phosphate contacts or participate in its dimer interface are indicated by pink boxes, magenta triangles below the E1 sequence, and green boxes, respectively. The information for E1 was obtained from the crystal structures of E1-obd with and without DNA.

Prokaryotic and viral origins contain multiple initiator binding sites. For DNA viruses, these binding sites consist of short DNA sequences, often organized as pairs of inverted repeats. The SV40 core origin is a 64-bp sequence that contains four such binding sites, termed P1 through P4 (collectively referred to as Site II). Each repeat has the sequence GAGGC. These pentameric sequences appear as a pair of inverted repeats, with a 1-bp spacer between each repeat (Figure 1A). The four GAGGC sequences are flanked by an early palindrome region on one side and an AT-rich region on the other side. There are, however, significant variations among viral origins in the spacing, the orientation within the origin, and the sequence of the binding sites. In the case of the related DNA tumor virus, bovine papilloma virus (BPV), the origin contains two pairs of imperfect repeats, and these are organized in a much more compact manner, such that the individual repeats overlap [7,8].

SV40 T-ag is a 708–amino acid protein containing at least three independent functional domains: an N-terminal J domain (amino acids 1–130), a central origin binding domain (obd) (amino acids 131–260), and a C-terminal helicase domain (amino acids 266–625). A flexible linker connects the obd to the helicase domain [9]. While there is no atomic resolution structure of the intact SV40 T-ag, structures of these individual domains are available. The crystal structure of the J domain has been solved in complex with retinoblastoma protein [10]. The crystal structure of the C-terminal helicase domain has been determined in the presence and absence of adenosine nucleotides [9,11] and with p53 [12]**.** Structural data of the T-ag obd in the absence of DNA include an NMR structure of a T-ag obd monomer [13] and a crystal structure of the T-ag obd in an open-ring form (spiral) having six subunits per turn [14].

Cryoelectron microscopy and biochemical studies of the full-length T-ag indicate that T-ag forms a “double donut” of hexameric rings in the presence of origin-like DNA and adenosine nucleotides [15,16]. In electron microscopy reconstructions, the J domains and the obds are near the center, and the helicase domains are at the distal ends of the intact dodecameric complex on DNA. The J domain is not required for replication in vitro (see [17,18] and references therein), and several lines of evidence suggest that the head-to head interaction of the hexameric rings is mediated by the obds and nearby residues [15,19]. The routing of DNA through the double hexamers is unclear, and none of the high-resolution structures of T-ag to date have included DNA. However, the recent structure of the BPV initiator E1 helicase domain shows that E1 forms a hexameric ring which contains single-stranded DNA (ssDNA) within its central channel [20]. “Rabbit ear” protrusions emanating from the dodecameric T-ag complex have been observed on electron microscopy, and these protrusions have been attributed to ssDNA coated by RPA [21]. Electron microscopic studies have also demonstrated considerable flexibility in the central region of the double hexamers where the obds are located [22–24].

T-ag has multiple functions, and the ability of the T-ag obd to transit between multiple modes of DNA binding and oligomerization states fits with the differing requirements of recognition, melting, and unwinding of DNA that must occur during DNA replication. The T-ag obd recognizes the GAGGC-containing duplex DNA at the origin and also binds double-stranded DNA (dsDNA) and ssDNA in a non–sequence-specific manner (reviewed in [1]). Previous biochemical experiments identified regions of the T-ag obd important in recognition of the GAGGC pentameric sequences, in particular, the A1 and B2 motifs [25] (amino acids 147–159 and amino acids 203–207, respectively). In addition, residues within these motifs also interact with ssDNA [26]. Moreover, regions of the T-ag obd (specifically, amino acids 167, 213, 215, and 220) participate in cooperative double-hexamer assembly in the context of the full-length T-ag [19]. Residues within the T-ag obd (amino acids 152–156, 181–182, 199–204, 255–258) also interact with other members of the replication machinery such as the C-terminal domain of human RPA32 [27] and RPA70AB [63].

Protein–DNA footprinting experiments have delineated the regions of the SV40 origin that are protected by T-ag. 1,10-Phenanthroline–copper footprinting data of DNA from the SV40 core origin complexed to either full-length T-ag or just the T-ag obd show similar protection patterns [28]. Such studies demonstrate that the DNA at P2 is protected by T-ag obd even when the P2 sequence is altered and that the DNA at P4 is less protected than sites P1 through P3, despite having the identical pentamer sequence. As assembly of double hexamers of T-ag on DNA requires only P1 and P3 [28], it appears that P2, and perhaps P4, is not essential for initial assembly in vitro. These data coupled with electron microscopic and mutagenesis data suggest that the obds bound to sites P1 and P3 could perhaps interact and guide subsequent assembly events.

Despite this wealth of biochemical and structural knowledge surrounding T-ag, it is unclear how the T-ag obd site-specifically recognizes the origin, whether DNA distortions are induced by this interaction, or how the obd participates in assembly of the double hexamer. Our recent crystal structure of the T-ag obd “spiral hexamer” [14] detailed the obd–obd interactions that occur upon formation of a single hexamer as well as the interactions between obds on opposing hexameric rings that could occur in the context of a double hexamer; however, it provided no insights into the T-ag obd–DNA interactions required for site-specific binding to the origin. To address these issues, we have solved two crystal structures of T-ag obds oriented head-to-head; with and without a DNA target.

The structures of four other DNA binding domains from viral initiator proteins have also been determined (reviewed in [29]), and although they share no apparent sequence homology with T-ag, the obds from SV40 T-ag [13,14], BPV E1 [30,31] and human papilloma virus E1 [32], the Rep proteins from adeno-associated virus 5 [33], and tomato yellow leaf curl virus [34] share a common fold. The SV40 T-ag is most closely related to BPV E1**,** but whereas SV40 large T-ag can bind to its origin DNA on its own, the BPV initiator E1 requires a loader or “matchmaker” protein, E2. Three crystal structures of the BPV E1 obd have been solved: the E1 obd dimer [30], the E1 obd dimer on DNA, and the E1 obd “tetramer” (two dimers) on DNA [31].

T-ag and E1 both form hexameric and double-hexameric helicase complexes on DNA, and their structural conservation suggests similarities in their mechanism of origin binding and helicase activity. However, there are significant differences in the architecture of these two viral origins. Thus, our structures of the SV40 T-ag obd have allowed us to differentiate aspects of origin recognition and helicase assembly that are specific to the individual viruses from those which are general and may be applicable to eukaryotic systems. Herein, we present the structural determinants of SV-40 origin recognition and a model of the structural rearrangements that accompany the transition from origin recognition of duplex DNA to formation of the dodecameric helicase.

## Results/Discussion

### Overview

In this paper we describe two crystal structures of the SV40 large T-ag obd: one in complex with duplex DNA and one as a dimer in the absence of DNA. The DNA oligomer used in the first crystallographic study contains two pentameric sites, P1 and P3, with P2 altered (Figure 1B). The second crystal structure is that of a T-ag obd dimer containing an intermolecular disulfide bridge between two Cys216 residues. Though the disulfide we observe may well be an artifact of crystallization, both of the structures reported here contain two T-ag obds arranged in a head-to-head orientation reminiscent of that seen in the structures of papilloma virus E1 obd. Thus, the subunits we see would presumably belong to opposing hexamers upon subsequent formation of double hexamers of large T-ag.

### Overall Structure of T-ag obd–DNA Complex

**Figure 2:**
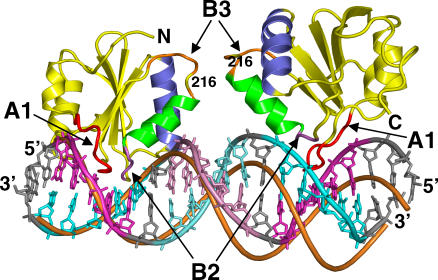
Overall Architecture of T-ag obds Bound to P1 and P3 of the Origin of Replication A ribbon diagram shows two T-ag obd monomers bound to the 21-mer DNA. The coloring of the DNA is the same as in Figure 1A. A ribbon diagram of an idealized B-form DNA is shown in orange superimposed on one binding site. The T-ag obd is colored as follows: red (A1 loop, amino acids 147–155), purple (B2 loop, amino acids 202–204), green (helix C), blue (helix B), orange (B3 loop amino acids 213–218), and yellow (Cys216 and the rest of the molecule). This orientation shows two T-ag obds arranged in a head-to-head fashion while engaged with DNA binding sites P3 and P1. This view shows A1 and B2 of each T-ag obd in the major groove of the DNA. All figures of molecules were generated with the molecular graphics program PyMOL [60].

**Figure 3:**
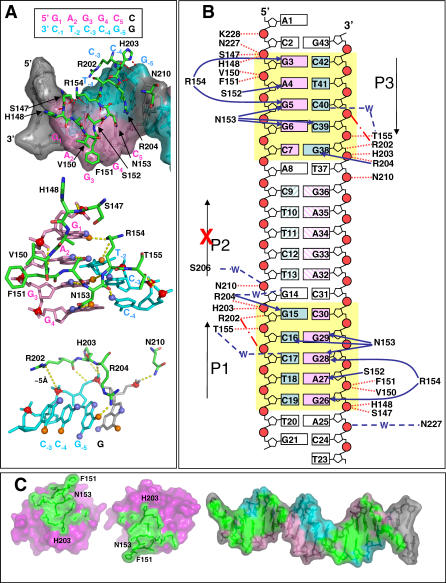
T-ag obd–DNA Interactions (A) Three close-up views of the protein–DNA interaction observed in the T-ag obd–DNA co-structure. In this figure, the GAGGC duplex is numbered and colored as shown in the boxed sequence. The top view shows both the A1 and B2 loops in the major groove of the DNA. The A1 and B2 loops are shown as sticks colored by atom type (carbon green, nitrogen blue, oxygen red). The DNA is shown as sticks with a translucent molecular surface and is colored as in Figure 2A. Red dashed lines indicate hydrogen bonds or electrostatic interactions. Residues are labeled. The middle and bottom views show the protein–DNA contacts from the A1 loop and the B2 loop, respectively. Atoms from the DNA involved in hydrogen bonds (yellow dashed lines) are shown as spheres: phosphate (red), oxygen (orange), and nitrogen (blue). (B) Schematic representation of protein–DNA interactions in the crystal structure. The DNA is numbered as shown. The GAGGC sequences of P1 and P3 are colored pink and their complements are colored cyan. The mutated P2 sequence is shown with the same coloring but with hash-marks. The P1 and P3 pentamers are placed in a yellow box. Arrows indicate the 5′ → 3′ direction of the GAGGC sequence. The red dotted lines indicate contacts with the phosphate backbone, and the blue solid arrows indicate sequence-specific H-bonds or salt-bridges. Blue dashed lines indicate observed water (W) mediated hydrogen bonds. (The dash-dot-dash line from Arg 202 indicates a phosphate interaction that could easily occur between the guanidinium group and a phosphate but is not observed in the structure.) (C) T-ag obd–DNA interface. The T-ag obd and DNA molecules are separated to show the interaction surface. Residues of T-ag obd which interact with DNA (and vice versa) are shown in green, and the rest of the T-ag obd molecule is magenta. The DNA is colored as in Figure 2A, but the surfaces which interact with T-ag obd are colored green. This exploded view clearly shows the two buried interaction surfaces (one for each T-ag obd) fill the major groove of the pentameric binding sites and that they occur on the same face of the DNA.

The crystal structure of the SV40 T-ag obd (amino acids 131–260) with duplex DNA containing two high-affinity binding sites, P1 and P3 (Figure 1), was refined to 2.4-Å resolution (Table 1). Pentanucleotide binding site P2 has been altered to abrogate site-specific binding. Longer DNA fragments having the same mutated P2 site as in our crystals have previously been shown to support assembly of double hexamers of T-ag [28,35]. The asymmetric unit contains two T-ag obd subunits and a DNA duplex 21 nucleotides long. The T-ag obd construct used in this study is shown in Figure 1D with the secondary structural elements and protein–DNA and protein–protein contacts indicated. In the crystal, the DNA stacks along its helical axis and forms a pseudo-continuous helix. The DNA oligomer is pseudo-palindromic, and the P1 and P3 binding sites can be considered as inverted repeats with a 7-bp spacer. The two T-ag obds are oriented head-to-head on approximately the same face of the DNA and make almost identical DNA interactions with their respective GAGGC sequences (Figures 2A and 3A). The obds are related by a pseudo 2-fold symmetry axis with a 171-degree rotation relating the two proteins. The DNA positions the obds such that the residues within the B3 loop (amino acids 213–220) are facing each other in an antiparallel fashion, with Phe218 from one monomer and Thr217 from the other are close to one another but not quite contacting. The electron density of the side chain of Phe218 is not clear, suggesting this side chain is flexible, and it could contact the second obd molecule in certain orientations.

**Table 1:**
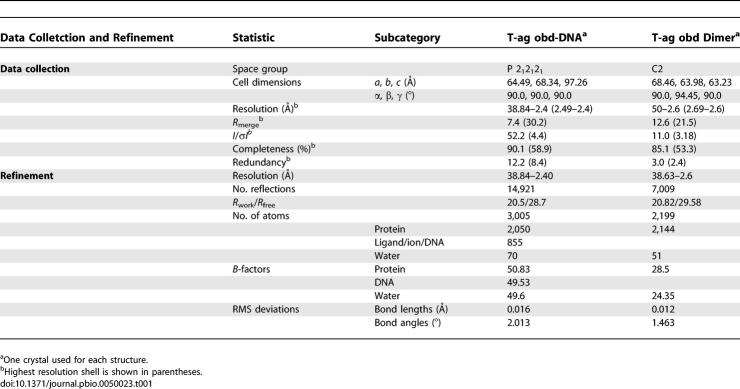
Data Collection and Refinement Statistics

### T-ag obd–DNA Interactions

Consistent with the observation that the nucleotides flanking the individual GAGGC sequences have little effect on binding affinity [36], all sequence-specific interactions in this crystal structure occur within the GAGGC sequence. Also in keeping with previous biochemical studies [37,38], each obd interacts with the DNA in the major groove primarily through the A1 (amino acids 147–159) and B2 (203–207) loops (Figures 2 and 3). A subset of residues within the A1 loop (amino acids 147–155) contacts both the phosphate backbone and the bases. Two residues within this motif, Asn153 and Arg154, make most of the base-specific interactions, with the pentanucleotide binding sites (P1 or P3). Residues adjacent or within the B2 loop (amino acids 202–204) interact primarily with the DNA phosphate backbone, with only Arg204 making sequence-specific interactions. For simplicity, we will continue to refer to the DNA binding loops as A1 (amino acids 147–155) and B2 (amino acids 202–204), although the precise definition of the residues within these loops differs somewhat from that described in the original biochemical work [25].

The site-specific binding of the T-ag obd to DNA buries approximately 1,600 Å^2^ per GAGGC pentamer (Figure 3C). This large buried surface area is consistent with the high affinities (*K_d_* of approximately 60 nm [36]) of the T-ag obd for the GAGGC sequence. The nucleotides in the structure are numbered in Figure 3B, but we will refer to a given nucleotide within the GAGGC (or its complement, GCCTC) by decreasing the font of the other nucleotides. For example, gAggc refers to the adenosine in position 2. The two residues from the A1 loop, Asn153 and Arg154, are situated deep in the major groove with the side chain of Asn153 extending toward the 3′ end and the side chain of Arg154 pointed toward the 5′ end of the GAGGC pentamer. Remarkably, these two residues interact with four of the five GAGGC nucleotides (GAGGc) in a sequence-specific manner through backbone and side chain interactions. Ser152 also makes sequence-specific contacts with gAggc (A27 or A4). The B2 loop residue Arg204 contacts the nucleotide Gcctc (G15 or G38) at both the base and the backbone. In terms of sequence specificity, both the N7 and O6 atoms (hydrogen bond acceptors) of the three guanines (GaGGc) participate in hydrogen bonds, explaining the importance of having a G at those positions. Indeed, two of these guanines have been shown to be essential (gaGGc) [39]. Conversely, only the N7 atom of the adenine (gAggc) accepts a hydrogen bond, suggesting that a guanine would also be tolerated at this position, as is the case in other polyomavirus origins [40]. Finally, both the N7 and O6 of the guanine on the complement strand (which base paired with the cytosine gaggC) participate in hydrogen bonds with Arg204, again, explaining a preference for a C-G base pair at this position (gaggC). There are no sequence-specific interactions between the obd and the altered P2 site.

The majority of the protein–DNA interactions from the A1 loop occur on the DNA strand that contains the sequence GAGGC. The protein–DNA interactions are summarized in a schematic in Figure 3B. In addition to the nucleotide-specific interactions, there are approximately ten hydrogen bonds and salt-bridges between the obd and nonbridging phosphate oxygen atoms per GAGGC sequence (Figure 3B). Most of these are from residues in the A1 loop (Ser147, His148, Val150, and Phe151) or the B2 loop (His203 and Arg204), but a few occur outside these loops (Asn210, Asn227, and Lys228). His203 has been previously shown to hydrogen bond with the phosphate backbone of GAGGC-containing dsDNA by NMR titration experiments [41]. Only one interaction is seen between a ribose oxygen O5′, and that occurs between Arg202 and Gcctc (G15 or G38). A number of van der Waals (ie, carbon–carbon) interactions (less than 4 Å) between the obd and DNA help stabilize the complex. Interestingly, most of these interactions occur between residues in the A1 motif (149, 151, 152, 153, 154, and 155) and with the base or the sugar carbons of the GAGGC-containing strand. van der Waals interactions occur outside of the GAGGC pentamer as well, at one nucleotide upstream of the pentamer Xgaggc (C2 or A25) and one nucleotide upstream of the complement pentamer Xgcctc (G14 or T37). Five water-mediated protein–DNA interactions (donor–acceptor distance less than 3.5 Å) are observed in the co-structure (Figure 3B). These interactions differ between the obds, and thus it is not clear that these are important specificity determinants.

The interaction of the two obds on P1 and P3 induces a 17-degree bend in the DNA. This bend allows the two obds to be significantly closer to one another than would be possible if the DNA were straight. Only a minor alteration in the DNA or protein structure would be needed for the odbs to interact with one another, and perhaps nucleate subsequent double-hexamer formation. The most severe distortions from canonical B-DNA are the compression of the minor groove and the phosphorous–phosphorous distance between the pentameric sequences P1 and P3 is 9.4 Å (versus 12.8 Å for standard B-form DNA). As changes from the natural sequence at site P2 could affect the DNA conformation, we cannot conclude that the native origin DNA is bent by the T-ag obd. We can, however, say with confidence that significant DNA deformation would be required for the T-ag obds to interact, a major departure from the picture presented in the structures of BPV E1 obd in complex with DNA derived from the BPV origin [31].

### T-ag obd–DNA Complex and E1 obd–DNA Complex Comparison

The BPV E1 origin also contains two inverted repeats (Figure 1C), but unlike the SV40 origin, the repeats in the BPV origin are overlapping and imperfect. This results in a much closer arrangement of the obds on their respective binding sites. Nonetheless, these two systems are grossly similar in the way they bind DNA. Both interact in the major groove via the same two loops. Both exhibit significant shape complementarity at the DNA–protein interface, and both obds use two adjacent residues splayed out in opposite directions to make most of their contacts within the major groove of the DNA (Asn153 and Arg154 in T-ag versus Lys186 and Thr187 in E1). The SV40 T-ag obd, however, makes more base-specific interactions than its BPV counterpart (wherein the only sequence-specific interactions are with the methyl group of thymine), and the SV-40–T-ag obd interactions are generally more electrostatic in nature. In addition, the T-ag obd engages both strands of the DNA to a greater degree than the E1 obds [31], as seen in the exploded view of the interaction surface (Figure 3C).

**Figure 4:**
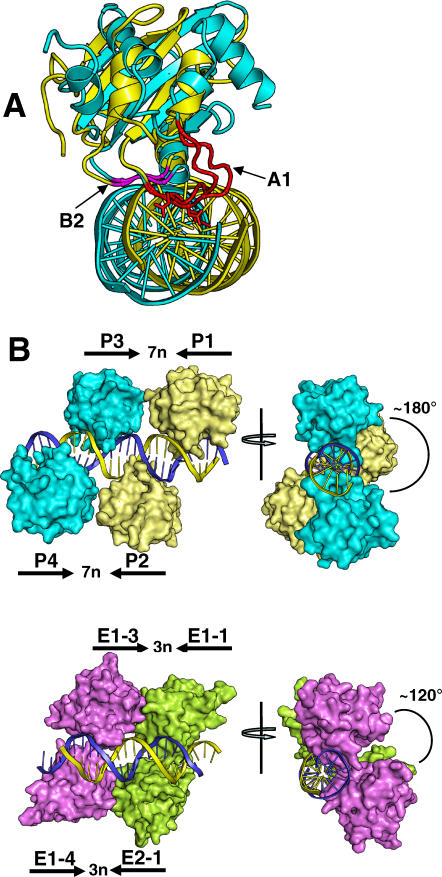
Comparison of T-ag obd and BPV E1 obd Co-Structures (A) The BPV E1 obd (cyan) was superimposed onto the T-ag obd (yellow). The molecules are displayed as ribbon diagrams looking down the helical axis of the DNA. The A1 and B2 loops (and their E1 equivalents) are shown in red and magenta, respectively. The residues that make most of the sequence-specific contacts in T-ag obd (Asn153 and Arg154) and the analogous E1 residues (Lys186 and Thr187) are shown as sticks. Part of the DNA in front of the A1 loop has been omitted from the figure. This view illustrates that even though the protein loops interacting with the DNA superimpose reasonably well, the DNA does not. (B) Comparison of the relative orientation of the SV40 and BPV obds bound to DNA. A model of four SV40 T-ag obds (yellow and cyan) engaged with the four GAGGC sites is shown. (Details of the construction of the model are in the legend for Figure 7.) The four BPV E1 obds (magenta and green) bound to the four E1 binding sites are shown below. The DNA is depicted as a ribbon diagram. The respective binding sites are labeled, and the number of nucleotides between the inverted repeats is shown. The T-ag obds do not interact, whereas the E1 obds form a dimer while bound to the inverted repeat E1–3 and E1–1 or E1–4 and E1–2. The T-ag obds bound to P1 and P2 (or P3 and P4) differ by approximately 180°, whereas the E1 obds bound to E1–1 and E1–2 (or E1–3 and E1–4) differ by approximately 120°. The two views, one looking down the helical axis of the DNA, illustrate the different spatial arrangement of the T-ag and E1 obds when bound to their respective *ori* sequences.

T-ag and E1 obds also differ in their orientation within the major groove of the DNA, and when one superimposes the SV40 and BPV obds, the respective DNA molecules do not overlay (Figure 4A). Conversely, superposition of the DNA molecules results in poorly superimposed obds. Differences also result because of the spacing of the binding sites. In the SV40 origin, the direct repeats (P1 and P2, or P3 and P4) are separated by one nucleotide and occur on opposite faces of the DNA, and the inverted repeats (P3 and P1 or P4 and P2) are separated by seven nucleotides and occur on the same face of the DNA (Figure 4B). In contrast, the analogous direct repeats in E1 overlap by three nucleotides, and the inverted repeats are separated by only three nucleotides (Figure 4B). Thus, it is not surprising that the E1 obds interact with each other upon binding DNA, whereas the T-ag obds do not. This difference in origin architecture is noteworthy because E1 dimerization upon the BPV origin is thought to be an important event in nucleation of the E1 double hexamer [42]. As discussed below, we believe that in the case of SV40, this dimerization event either occurs later in the assembly process, when the obds are no longer engaged with the GAGGC sequence, or is accompanied by significant DNA deformation.

The dissociation constant of the T-ag obd for DNA containing both pentamers P1 and P3 is 60 nM, very similar to that for a single GAGGC sequence within a larger DNA oligomer (*K_d_* = 57 to 150 nM) [36]. This is in contrast to the much weaker affinity of the BPV E1 obd for a single site (*K_i_* = 517 nM) and a comparable affinity for two correctly spaced E1 sites (32 nM) [43]. Consistent with its more numerous DNA contacts, the SV40–T-ag obd–DNA interaction buries a larger surface area (approximately 1,600 Å^2^ per obd–GAGGC interaction, shown in Figure 3C) than the analogous E1 obd–DNA interaction (approximately 1,000 Å^2^ for E1/ATTGTT). This could help explain the higher affinity of T-ag obd for its DNA target site. In addition, T-ag obd binds approximately 10-fold more tightly to its specific binding site than to random DNA [36], whereas E1 binds less than 2-fold more tightly [43]. These data may also explain why DNA binding by T-ag obd is more specific than that of the E1 obd and why E1 requires a helper protein (E2) to load it onto the DNA and T-ag does not.

### Structure of the T-ag obd Dimer

**Figure 5:**
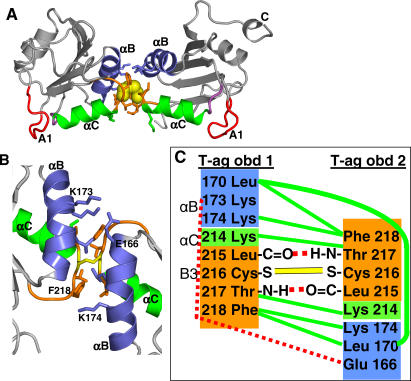
T-ag obd Disulfide-Linked Dimer Structure (A) T-ag obd dimer. Ribbon diagram of the T-ag obd dimer an orientation similar to Figure 2A. The A1 (red), B2 (purple), B3 (orange) loops, helix αB (blue), and helix αC (green) are colored as in Figure 2A. Cys216–Cys216 disulfide linkage is shown as yellow van der Waal spheres. (B) A ribbon diagram of a close-up of the dimer interface using the same coloring as in (A). Side chains of residues that participate in the interface are shown. (C) Schematic of dimer interface. The residues (less than 4 Å apart) involved in the dimer interface are shown. The disulfide bond is shown as a yellow line connecting the two Cys residues. The hydrogen bonds are indicated as red dashed lines, and van der Waals (carbon–carbon) interactions are depicted by solid green lines.

The second crystal structure we report is that of a T-ag obd dimer in the absence of DNA. This structure has been refined to 2.6-Å resolution (Table 1). The asymmetric unit contains two T-ag obd molecules linked together by a disulfide bond. Although the presence of the disulfide bond is likely an artifact of crystallization, we include it here because it facilitates our description of structural changes associated with DNA binding. Perhaps coincidentally, the obds in this dimer are oriented in a head-to-head fashion and contact one another using the same loops which mediate the inter-obd contacts in the structures of BPV E1 (Figure 1B). As shown in Figure 5A, the monomers are related by a pseudo 2-fold symmetry axis with a rotation of 178° between the molecules. The dimer interface contains a mixture of hydrophobic and hydrophilic interactions and buries a surface area of approximately 740 Å^2^. For comparison, the E1 obd dimer interface, an interface which is seen in crystal structures with and without DNA, buries only approximately 500 Å^2^. The T-ag obd–obd interface is nearly symmetric with almost identical residues (18 total) from each monomer contributing atoms to the interaction surface. These residues are from helix αB (Glu166, Leu170, Lys173, and Lys174), residues at the end of helix αC, and residues from the B3 loop (amino acids 213–218) (Figure 5B and 5C). Interestingly, T-ag mutants within the B3 loop (Q213H, L215V, and F220Y) are impaired in their ability to form double hexamers, and mutation of other residues nearby (K167R and A168V) is impaired in both double-hexamer formation and unwinding duplex DNA [19]. In addition, the cysteine residue bridging the two obds (Cys216) is completely conserved across the Polyoma virus family, and the C216G mutation in T-ag has been shown to be defective in unwinding closed circular DNA [44]. In summary, although the existing literature clearly indicates that the residues at the protein–protein interface observed in the disulfide-linked dimer are important for T-ag assembly and helicase function, this similarity could be coincidental. Furthermore, while we believe that something like the dimeric structure we observe may well be important for stabilization of the T-ag double hexamer, the structure we present cannot be considered evidence of this.

### Changes in obd Conformation upon DNA Binding

**Figure 6:**
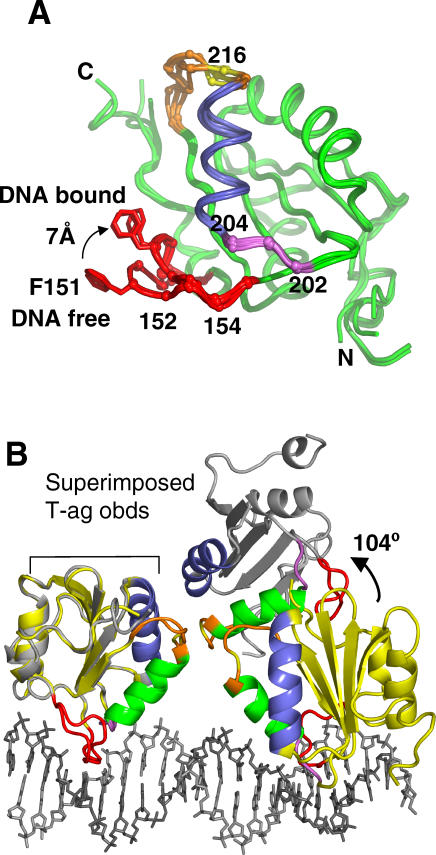
Superposition of T-ag obd Monomers (A) The five nonequivalent crystallographically observed T-ag obd monomers (two from the co-structure, two from the dimer structure, and one from the spiral hexamer structure) are superimposed and displayed in a tube representation. The coloring is as follows: A1 (red), B2 (purple), B3 loop (amino acids 215–220, orange), Cys216 (yellow), and everything else (green). The side chain of Phe151 is shown to indicate the range of motion of A1 loop. The DNA-free (“up”) and DNA-bound (“down”) positions of the A1 loop are indicated. (B) Comparison of T-ag obd co-structure and disulfide-linked dimer structure. A superposition of the T-ag obd dimer onto the T-ag obd co-structure is displayed as a ribbon diagram. Loops A1, B2, and B3, helix αB, and helix αC are colored as above. The rest of the T-ag obd in the co-structure is colored yellow; the rest of the disulfide-linked dimer is gray. One T-ag obd monomer of the disulfide-linked dimer (gray) was superimposed on one T-ag obd monomer (yellow) from the co-structure. The superimposed monomer is shown on the left. The relative orientation of the second monomers differs by 104°.

Our previously published crystal structure of T-ag obd in the absence of DNA showed an open-ring conformation having six obds per turn [14]. Together with the two crystal structures presented here, each with two copies of the obd in the asymmetric unit cell, we now have five crystallographically independent structures of T-ag obd monomers for comparison. Interestingly, the B2 loop, which makes the majority of the DNA contacts, is virtually identical with and without the DNA (Figure 6A). Pairwise least-squares superpositions of these T-ag obd monomers reveal root-mean-square deviations in Cα positions of 0.4–1 Å. The superposition, shown in Figure 6A, reveals that the most dramatic difference in the structures occurs in the A1 loop and that the amino acid that varies most is Phe151 (approximately 4-Å Ca–Cα distance, approximately 7-Å tip–tip distance). Although there are five crystallographically independent molecules, only two conformations are seen. The two obds from the DNA complex structure have the A1 loop in one orientation (flipped “down”), while the three obds crystallized in the absence of DNA have the A1 loop in another conformation (flipped “up”). There is no steric clash of the A1 loop that would force this change in conformation (from “up” to “down”) upon binding DNA. Rather, shape and charge complementarity appear to favor the “down” orientation in the presence of DNA. Phe151 comprises an integral portion of the protein–protein interface observed in the spiral structure and perhaps plays a role in the structural reorganization of the obds from origin recognition to oligomerization. Interestingly, in the portion of the A1 loop that provides sequence-specific interactions, namely Asn153 and Arg154, the position of the Cαs hardly changes between the DNA-bound and DNA-unbound forms. This indicates that the sequence-specific determinants for DNA binding are preformed in the absence of DNA. The residues in loop B3 also exhibit some differences among the three structures, but the electron density in this region was poor in all structures except the disulfide-linked one.

### Relative Orientation of T-ag obd Monomers

**Figure 7:**
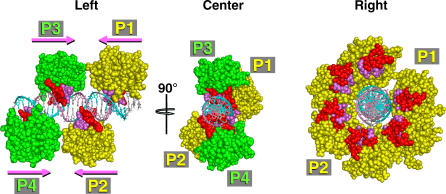
Modeling Studies of the T-ag obd A model of four T-ag obds engaged with DNA containing P1 through P4. Starting with the x-ray coordinates of the co-structure of two T-ag obds on P1 and P3, a model was generated of four T-ag obds engaged with the four pentameric binding sites P1–P4. As stated in the text, assembly of the double hexamer of T-ag does not require all four pentanucleotides, although all four are required for unwinding. However, given that the origin contains four pentanucleotides and the structure does not predict any steric clashes when all four are sites are occupied, it is likely that all four are occupied by the obd at some point during assembly. The DNA is colored as Figure 2A. The T-ag obds are shown as van der Waals spheres. The obds that engage with P1 and P2 will presumably comprise one hexamer (yellow). The obds that engage with P3 and P4 will presumably comprise the second hexamer (green). The A1 and B2 loops that engage with the DNA are colored red and purple, as in Figure 2A. The 5′ → 3′ direction of the GAGGC sequences is indicated by arrows and labeled. (Left) In this view, the obds bound to P1 and P3 (or P2 and P4) are oriented head-to head. As stated in the text, obds bound to P1 and P3 (or by extension, P2 and P4) are close but do not contact. This model also shows that the obds on adjacent pentamers (P1 and P2 or P3 and P4) do not interact with each other. (Center) This view is looking down the axis of the DNA and shows clearly that the obds bound to adjacent pentamers occur on opposite faces of the DNA. (Right) This is a view of spiral hexamer of T-ag obd, with the DNA interacting loops colored the same as in the other panels. Duplex DNA is modeled along the central channel of the spiral. The obds that are 180° apart and could have previously interacted with binding sites (eg, P1 and P2) are indicated. This figure illustrates two important points. First, the approximately 30-Å-diameter channel of the spiral hexamer positions the obds farther from the DNA than when engaged site-specifically. Second, the position of the DNA binding loops in red and purple indicates that a significant rotation of the obds must occur to transit from the sequence-specific DNA binding structure to the spiral structure.

While the structures of the individual monomers of T-ag obd are very similar, there are significant differences in the relative orientation of the monomers in the two crystal structures reported here. Both the co-structure and the dimer structure are oriented in a head-to-head fashion with the B3 loops pointed toward one another, but when one superimposes one monomer of the disulfide-linked dimer onto a DNA-bound monomer, the second set of monomers differ in orientation by 104° (Figure 6B). The molecular orientations in these two structures also differ significantly from that seen in the spiral ring of obd subunits, and from our model of the head-to-head interaction of these spirals. These differences reinforce our prediction that the T-ag obd spiral seen in the previous crystal structure of this domain cannot exist at the same time as the T-ag obd–DNA–specific complex. If the DNA travels down the center of the spiral structure, the A1 and B2 loops in the spiral are neither close enough nor oriented properly to engage the GAGGC sequences as seen in the co-structure (Figure 7, right). Significant structural rearrangement would be required, and the consequences of these rearrangements are considered below.

### A Model of T-ag Assembly and DNA Threading

**Figure 8:**
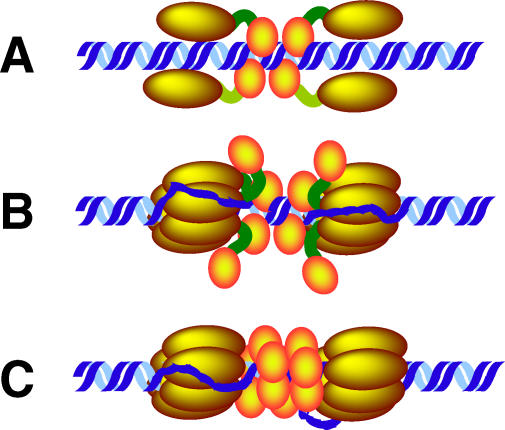
Schematic of SV40 T-ag Assembly on Origin DNA The SV40 origin dsDNA is depicted as two ribbons. The SV40 T-ag N-terminal J domain is omitted for clarity. The SV40 T-ag domains are depicted as follows: the obd (yellow spheres), the helicase domain (yellow ellipsoids), and the flexible linker that connects them (green). (A) The T-ag obd binds its high-affinity GAGGC sites. The T-ag obd anchors the protein on the four GAGGC pentamers and thus orients the helicase domain for appropriate DNA strand selection in the subsequent steps. The obds on P2 and P4 are shown as transparent spheres to indicate that they are not crucial for single-hexamer formation but are required for unwinding. The helicase domains may interact as monomers with the DNA in a non–sequence-specific manner at this point. (B) Once the origin has been recognized by the obds, the two helicase domains each hexamerize around one strand of DNA. As a result, one strand goes through the central channel of the helicase domain, and the other traverses the surface of the helicase domain. This is consistent with the crystal structure of the E1 helicase domain with ssDNA [20], and this model is similar to that proposed for other hexameric helicases [50]. It is not known whether the DNA at site II is melted at this point or not. Twelve obds are now in close proximity and may now interact with one another despite relatively weak affinities. (C) Interaction between the two hexamers could occur through a series of obd–obd structural rearrangements wherein these domains transition from the site-specific complex with the A1 and B2 loops fully engaged with the DNA, to a structure where loop B3 makes contacts across a pair of obds (possibly as spirals). The A1 and B2 loops are now oriented away from the central channel (and are proximal to the helicase domains). The open ring spiral hexamer of the T-ag obd would allow access of ssDNA from the outside surface of the helicase domain to the center of the channel. This channel is positively charged and sufficiently wide (approximately 30 Å) to accommodate two ssDNA strands moving in opposite directions. It is likely that the obds are dynamic and fluctuate between differing states of interaction, including aclosed hexameric ring, depending on the requirement to interact with ssDNA, dsDNA, or other factors, such as the SSB hRPA. The double hexamer is now assembled and ready to recruit other host factors necessary for replication (for a review, see [3]).

In this paper we present crystal structures of the SV40 T-ag obd in the presence and absence of DNA. Together, with the previously solved high-resolution “spiral hexamer” of T-ag obd, these results provide a structural framework upon which to describe the molecular events require for initiation of SV40 DNA replication. Formation of the helicase-competent T-ag–DNA complex involves at least four molecular events: monomer recognition of the dsDNA at the origin, assembly of hexamers and double hexamers on DNA, DNA melting, and threading of the DNA through the T-ag complex. Although the sequence of these events remains unclear, and some steps may occur simultaneously, the extensive literature on T-ag and related systems allows us to propose a temporal context for the crystal structures presented here (Figure 8).

In our model, the initial step in origin recognition involves formation of a complex very similar to that seen in our DNA co-structure. Although the helicase domain can bind DNA [45–47], only the obds contain significant nucleotide sequence specificity, and it is thus reasonable to propose that binding of individual obds to individual GAGGC binding sites occurs first in the assembly process. As suggested by earlier studies involving T-ag (reviewed in [1]) and those involving papillomavirus E1 [31], the T-ag obds occupying P1 and P2 would ultimately belong to one hexamer, while those occupying P3 and P4 would belong to the other hexamer (Figure 7). A single pentameric sequence is statistically likely to occur once every 512 base pairs [(4^5^)/2] and does not in itself provide much selectivity. Two correctly spaced pentamers should, however, occur only once every 500,000 base pairs. Consistent with this idea, an individual GAGGC sequence supports single-hexamer formation of T-ag [28], but occupancy of at least two correctly spaced binding sites (eg, the inverted repeats P1 and P3) is required for double-hexamer formation [28,35,48].

Within a single hexamer, the dominant T-ag–T-ag interaction likely occurs through the helicase domains (an interaction that buries 4,344 Å^2^ in the presence of ATP [11]). Whereas these domains readily form hexamers in the absence of the obd [49], isolated obds have little propensity to interact with one another in solution, and in the crystal structure of the obds arranged in a 6-fold symmetric spiral, the buried surface area between these domains is only 1,300 Å^2^ [14]. Nonetheless, mutation of residues within the obd at positions F183 and S185 disrupts formation of T-ag hexamers, suggesting that the obds are also important to the integrity of the hexameric complex [50]. Both of these residues occur at or near the T-ag obd–obd interface seen in the open-ring obd structure [14] and both are far from the DNA-binding interface. Mutation of residues in the B3 loop, another region far from the DNA, also impairs double-hexamer formation [19]. Thus, several lines of evidence suggest that interaction among obd subunits may be important for the integrity of the double-hexamer T-ag complex. As described above, we believe that the first step in origin recognition involves the binding of obd subunits to unmelted GAGGC pentamers, and in the co-structure presented here, obds do not interact with one another while bound to DNA. This is consistent with the observation that isolated obds exhibit no cooperativity in their DNA binding [36]. Thus, while interactions among the obds may be important later in the assembly process, interaction among these domains (in either single- or double-hexamer formation) does not seem likely during the very early stages of assembly.

The model in which the obds bind to their respective GAGGC sites before other assembly events is attractive in a number of respects, most importantly, because it suggests an explanation for how the DNA is threaded through the T-ag double hexamer. In this model, double-hexamer assembly and DNA threading occur simultaneously. The obd of T-ag serves to anchor and orient the complex at a distinct location on the DNA, and strand selection occurs as a consequence of this location, the nature of the protein–DNA interactions, and the dynamics of the spontaneous ring formation of the helicase domains. Similar models have been presented (reviewed in [51]); however, given the structures presented here and recent developments in our understanding of helicase domain–ssDNA interactions [20,26,47], we believe these models need some modification. As pointed out by Enmark et al. [20], the diameter of the central channel of the T-ag helicase domains is too small to accommodate dsDNA, but it can accommodate ssDNA. Thus, we believe that DNA strand separation occurs as a consequence of the hexamerization of the helicase domains around a single DNA strand. Binding of a single strand is supported by the crystal structure of the BPV E1 helicase domain in complex with ssDNA [20], and our model of SV40 assembly is also in line with the steric exclusion mechanism used by other hexameric helicases such as the Escherichia coli transcription termination factor Rho [52].

Once the proper strands have been selected and assembly of the double hexamer is under way, T-ag must release the double-stranded GAGGC binding sites to which it is attached. In our model, once the obds are no longer needed for origin recognition, they transition into a double-ring structure, and we believe this structure helps to hold together the T-ag double hexamer**.** This model positions the amino acids in helix αB (K167) and in loop B3 (Q213, L215, and F220), which when mutated result in defects in double-hexamer formation [19], opposite one another on two head-to-head rings composed of obds, and is consistent with electron microscopic images showing the obds at the hexamer–hexamer interface [15].

We envision that the DNA containing the pentamers is melted at this point, with opposing strands passing through each of the two helicase domain rings. The diameter of the inner channel (approximately 30 Å) of the T-ag obd spiral hexamer crystal structure is sufficiently large to accommodate either dsDNA or two single strands of DNA. In the obd spiral structure, the DNA-binding regions (the A1 and B2 loops) are rotated away from the DNA axis and thus can no longer engage the pentamers in a sequence-specific manner. This structural rearrangement explains how the same residues on the T-ag obd can be responsible for both base-specific DNA recognition of the duplex and nonspecific duplex and ssDNA binding [26].

It has been shown that assembly of the double hexamer of T-ag causes DNA strand separation of the early palindrome region (reviewed in [6]) and, presumably, melting of the AT flanking sequences would follow. If the assembly of the double hexamer of T-ag causes DNA strand separation on either side of Site II (within the flanking sequences), the structural transition of the obds from origin recognition to formation of a hexameric ring could be promoted by melting of the DNA within the obd binding sites. The high local concentration of obds resulting from formation of this dodecameric complex also might be expected to shift the equilibrium in favor of ring formation of the obds, despite their weak propensity for self-association (Figure 8).

Many of the same residues of T-ag obd that bind the DNA have also been shown to bind the ssDNA binding-protein human RPA [27], an interaction that is likely to cause steric clashes in the spiral hexamer unless one or more of the obds rotate out from the central ring so as to more fully expose their A1 and B2 loops. While the spiral already provides a “gap” for the ssDNA strand to exit the ring, such a rotation of obds away from the DNA would allow both easier access for accessory proteins such as hRPA and easier egress for ssDNA. This model suggests that the region in the center of the T-ag double hexamer is dynamic and would lack the distinct, 6-fold symmetric symmetry present in the helicase domains, a picture consistent with the results of recent single particle analysis of T-ag on DNA [24].

### Conclusion

In conclusion, the various structures of the SV40 T-ag obd on and off its DNA target have delineated the atomic determinants of DNA binding and have allowed us to propose a model of the rearrangements that the obd undergoes as T-ag progresses from origin recognition to formation of the dodecameric complex. Despite the gross similarities between SV40 T-ag and BPV E1, there appear to be some significant differences in the modes of assembly between the two systems. First, the BPV E1–E1 dimer interface has been shown to be important for E1 to bind its DNA target [30]. Interaction between SV40 T-ag obds on DNA is not observed in our crystal structure, and such interactions cannot occur without significant DNA deformation. Second, the BPV E1 is thought to form head-to-head double trimers on DNA prior to forming double hexamers [42]. In BPV, the obd-binding sites analogous to P1 and P2 are separated by approximately 120° along the DNA helical axis, and it is easy to see how a trimer of obds on DNA might form. The architecture of the SV40 origin, however, places sites P1 and P2 roughly 180° apart. Thus, from a structural standpoint, a 3-fold symmetric intermediate of T-ag on DNA is hard to justify. Furthermore, biochemical studies suggest that T-ag forms only monomers, hexamers, and, in the presence of an appropriate DNA, double hexamers [49].

Recent progress has provided atomic-resolution pictures of a number of key interactions among T-ag domains and with DNA. Among these are the interaction between obd monomers that facilitate their assembly into open rings containing a 30-Å inner diameter [14], the interactions between the helicase domain subunits that allow these domains to assemble into 6-fold symmetric machine that couples ATP hydrolysis to DNA translocation [9,11], the interactions between the related BPV E1 helicase rings and ssDNA [20], and the interactions between the obds and dsDNA that explain how origins are recognized. While some aspects, most notably, the determinants holding together the double hexamers, remain uncertain, this collection of high-resolution structures has allowed us to develop very specific predictions which can now be probed experimentally to test and refine our understanding of this complex system.

## Materials and Methods

### Protein expression and purification.

The SV40 T-ag obd (amino acids 131–260) was overexpressed in E. coli as a GST-fusion and purified as previously described [14]. The purified protein was dialyzed into storage buffer (10 mM Tris [pH 7.5], 50 mM NaCl, 10% glycerol, 1 mM DTT), concentrated by ultrafiltration using a VivaSpin 500 (VivaScience, http://www.vivascience.com), aliquoted and flash-frozen in liquid nitrogen, and stored at −80 °C.

### DNA purification.

Synthetic DNA oligonucleotides were synthesized leaving the trityl group on by the phosphoramidite method (Keck Facility, Yale University, New Haven, Connecticut, United States). The oligomers were cleaved and deprotected while in the cartridge. The oligomers were detritylated and purified in a single step using a semipreparative DNAPure column (Rainin Instruments, http://www.rainin.com). Oligomers were lyophilized to dryness and resuspended in 10 mM Tris (pH 7.5), 50 mM NaCl.

### DNA duplex formation and crystallization.

Duplex DNA was formed by mixing a 1:1 ratio of complementary oligomers in annealing buffer (10 mM Tris [pH 8], 50 mM NaCl) based on the calculated extinction coefficient at 260 nm. The concentration of DNA was approximately 0.1 mM. The mixture was heated in a water bath to 94 °C and allowed to cool slowly over several hours to 4 °C. The duplex DNA was stored at −20 °C until ready for use.

The T-ag obd–DNA complex was prepared in a 2:1.1 molar ratio by slowly adding duplex DNA (approximately 0.1 mM) to the T-ag obd (approximately 5 to 8 mg/ml). The resultant mixture was further concentrated to a T-ag obd concentration approximately 20 mg/ml by ultrafiltration using a VivaSpin 500 (VivaScience). The complex was flash frozen in liquid nitrogen and stored at −80 °C. Crystals of T-ag obd in complex with the 21-mer duplex DNA were grown at 4 °C under paraffin oil in sitting drops using a microbatch optimization strategy [53]. From 3 to 6 μl of crystallization solution (0.12 M sodium cacodylate [pH 6.5], 0.24 M calcium acetate, 14% v/v PEG 8000) were mixed with 5 μl of the T-ag obd–DNA complex in a 150-μl PCR tube. This mixture was placed in a sitting drop tray under paraffin oil. Crystals grew in approximately 5 d as thin plates.

Crystals of the T-ag obd dimer (in the absence of DNA) were grown by vapor diffusion using the hanging drop method at 20 °C. Then 1 μl of the T-ag obd (8.8 mg/ml) in storage buffer was mixed with 1 μl of a reservoir solution consisting of 30% PEG 4000, 0.1 M sodium citrate (pH 5.6), and 0.2 M ammonium acetate. The drop was equilibrated over a 0.4 ml reservoir solution. Crystals grew in approximately 1 wk.

### Structure determination and refinement.

For the DNA complex, single crystals were harvested and slowly transferred to a final cryogenic solution (0.1 M sodium cacodylate [pH 6.5], 0.1 M calcium acetate, 30% v/v PEG 8000, 20% glycerol) and flash-frozen in LN2. Data to 2.4 Å were collected at Beamline X29 at the National Synchrotron Light Source (Brookhaven, New York, United States) at a wavelength of 1.1 Å, at 100K, and using a Quantum 315 detector. The data were processed with HKL2000 [54] and scaled with SCALA [55].

A molecular replacement search model based upon coordinates of a T-ag obd in complex with a 5-bp duplex GAGGC (Alexey Bochkarev, personal communication) was constructed. Molecular replacement was performed with the program PHASER [56] in all primitive orthorhombic space groups. PHASER identified the space group as P2_1_2_1_2_1_ and positioned two molecules of the T-ag obd in the asymmetric unit. The missing DNA was visible in the resulting electron density map and was built using the molecular graphics program COOT [57]. Although the protein in these crystals has a unique orientation, the DNA can be positioned in two different orientations without changing the R-factor. As the DNA sequence is pseudo-palindromic, this static disorder in our crystals had no deleterious effect on the quality of the electron density at the GAGGC repeats or at the DNA phosphate backbone. The density for the bases outside of the protein-binding sites, however, was equally consistent with either of the two possible DNA orientations. As attempts to model both DNA orientations simultaneously (each with half occupancy) did not significantly reduce either the working or the free R-factor, only one of the two orientations is present in our final model of the DNA–protein complex. In addition, no *sequence-specific* protein–DNA contacts occur outside the GAGGC sequences. Multiple rounds of building and simulated annealing were performed with the program CNS [58] or REFMAC [59]. A simulated annealing omit map is presented in Figure S1. The final rounds of refinement included TLS refinement. The final model consists of two molecules of T-ag obd, one 21-mer duplex DNA, and 70 water molecules. The final R-factor and R-free are 20.5% and 29.0% (from REFMAC). Refinement statistics for the final 2.4-Å model are summarized in Table I.

For the T-ag obd dimer crystal, single crystals were harvested and slowly transferred to a final cryogenic solution (0.1 M sodium citrate [pH 5.6], 0.2 M ammonium acetate, 30% PEG 4000, 20% glycerol) and flash-frozen in LN2. Data to 2.6 Å were collected at Beamline X29 at the National Synchrotron Light Source at a wavelength of 0.9791 Å, at 100K, and using a Quantum 315 detector. The data were processed with HKL2000 and scaled with SCALA. The crystals were characterized as having the space group C2 with two molecules in the asymmetric unit. A molecular replacement search model from the x-ray coordinates of the T-ag obd was made. The structure was solved by molecular replacement using the program PHASER. The resulting electron density map showed clear density for a disulfide bridge between the two monomers. The model was built and refined using the molecular graphics program COOT. Several rounds of building and simulated annealing were performed with the program CNS or REFMAC. The final model consists of two molecules of T-ag obd and 51 waters. The final R factor and R_free_ values are 20.82% and 29.58% (from REFMAC). Refinement statistics are summarized in Table I.

Figures were made using the molecular graphics program PyMOL [60]. The DNA structure was analyzed using the programs 3DNA [61] and MADBEND [62].

## Supporting Information

Figure S1 Simulated Annealing Omit Map of a GAGGC Duplex To remove phase bias, this region of our model (a GAGGC duplex) was removed and the remaining atoms were subjected to simulated annealing refinement in CNS prior to map calculation. The electron density of the F_o_ − F_c_ map is contoured at 2σ. The GAGGC strand is colored pink and its complement is cyan.
(565 KB PPT)

### Accession Numbers

Protein Data Bank (http://www.rcsb.org/pdb) accession numbers for the coordinates for the T-ag obd co-structure and disulfide-linked dimer are 2NTC and 2IF9, respectively, and for the T-ag obd residues that comprise the protein–protein interface in the spiral hexamer, 2FUF. The information for E1 was obtained from the crystal structures of E1-obd with and without DNA (Protein Data Bank accession numbers 1F08, 1KSY, and 1KSX).

## Abbreviations

BPV: bovine papilloma virus
dsDNA: double-stranded DNA
obd: origin-binding domain
RPA: replication protein A
SSB: single-stranded binding protein
ssDNA: single-stranded DNA
SV40: simian virus 40
T-ag: T-antigen

## Note Added in Proof

Reference 63 is cited out of order in the article because it was added while the article was in proof.
